# Exposure to a Patient-Centered, Web-Based Intervention for Managing Cancer Symptom and Quality of Life Issues: Impact on Symptom Distress

**DOI:** 10.2196/jmir.4190

**Published:** 2015-06-03

**Authors:** Donna L Berry, Traci M Blonquist, Rupa A Patel, Barbara Halpenny, Justin McReynolds

**Affiliations:** ^1^ Phyllis F. Cantor Center Dana-Farber Cancer Institute Boston, MA United States; ^2^ Department of Medicine Harvard Medical School Boston, MA United States; ^3^ Biostatistics & Computational Biology Dana-Farber Cancer Institute Boston, MA United States; ^4^ Usability Engineering GoDaddy Group Kirkland, WA United States; ^5^ Phyllis F. Cantor Center Dana-Farber Cancer Institute Boston, MA United States; ^6^ Biomedical and Health Informatics University of Washington Seattle, WA United States

**Keywords:** patient-centered technology, cancer, symptoms, quality of life, eHealth, Internet

## Abstract

**Background:**

Effective eHealth interventions can benefit a large number of patients with content intended to support self-care and management of both chronic and acute conditions. Even though usage statistics are easily logged in most eHealth interventions, usage or exposure has rarely been reported in trials, let alone studied in relationship to effectiveness.

**Objective:**

The intent of the study was to evaluate use of a fully automated, Web-based program, the Electronic Self Report Assessment-Cancer (ESRA-C), and how delivery and total use of the intervention may have affected cancer symptom distress.

**Methods:**

Patients at two cancer centers used ESRA-C to self-report symptom and quality of life (SxQOL) issues during therapy. Participants were randomized to ESRA-C assessment only (control) or the ESRA-C intervention delivered via the Internet to patients’ homes or to a tablet at the clinic. The intervention enabled participants to self-monitor SxQOL and receive self-care education and customized coaching on how to report concerns to clinicians. Overall and voluntary intervention use were defined as having ≥2 exposures, and one non-prompted exposure to the intervention, respectively. Factors associated with intervention use were explored with Fisher’s exact test. Propensity score matching was used to select a sample of control participants similar to intervention participants who used the intervention. Analysis of covariance (ANCOVA) was used to compare change in Symptom Distress Scale (SDS-15) scores from pre-treatment to end-of-study by groups in the matched sample.

**Results:**

Radiation oncology participants used the intervention, overall and voluntarily, more than medical oncology and transplant participants. Participants who were working and had more than a high school education voluntarily used the intervention more. The SDS-15 score was reduced by an estimated 1.53 points (*P*=.01) in the intervention group users compared to the matched control group.

**Conclusions:**

The intended effects of a Web-based, patient-centered intervention on cancer symptom distress were modified by intervention use frequency. Clinical and personal demographics influenced voluntary use.

**Trial Registration:**

Clinicaltrials.gov NCT00852852; http://clinicaltrials.gov/ct2/show/NCT00852852 (Archived by WebCite at http://www.webcitation.org/6YwAfwWl7).

## Introduction

### Background

Clinicians and researchers have developed eHealth solutions that supplement the limited time for patient report and communication within the confines of the ambulatory care, face-to-face visit [1]. Reported benefits of eHealth solutions for oncology care include improved patient well-being [2,3], better patient-clinician communication [2,4], and lower symptom distress [5]. Effective eHealth interventions can benefit a large number of patients with both generic and tailored content. Even though usage statistics are easily logged in most eHealth interventions, usage or exposure has rarely been reported in trials, let alone studied in relationship to effectiveness. As reviewed by Donkin et al [6], the “dose” of eHealth solutions, comprehensive measures of intervention exposure or patient engagement, have been documented in few trials evaluating health promotion and mental health interventions. Furthermore, eHealth intervention delivery has been studied in only one cancer symptom and quality of life trial, in association with outcomes in breast cancer survivors [7]. The ability and efforts of patients in active cancer treatment to fully utilize such solutions are uncertain.

The Electronic Self Report Assessment for Cancer (ESRA-C) is a patient-centered technology developed with rigorous participatory design methods [8] and evaluated in multi-site randomized trials [4,5]. ESRA-C, a Web-based intervention that supports patients with any cancer diagnosis during treatment, has been shown to significantly increase the frequency of patient-clinician communication about problematic issues [4], reduce symptom distress over the course of active therapy [5], and increase the patient’s unsolicited and specific description of symptoms and quality of life (SxQOL) concerns [9]. However, when we conducted a mediation analysis of the impact of the intervention group’s increased patient verbal reports at one clinic visit during the trial, we found no significant impact on the primary outcome of symptom distress [9]. In other words, another aspect of the intervention was responsible for the reduction of symptom distress.

### Objective

The purpose of this analysis was to determine the impact of the ESRA-C intervention exposure on cancer symptom distress and describe frequency of intervention use by participants in the ESRA-C II trial.

## Methods

### Overview

**Figure 1:**
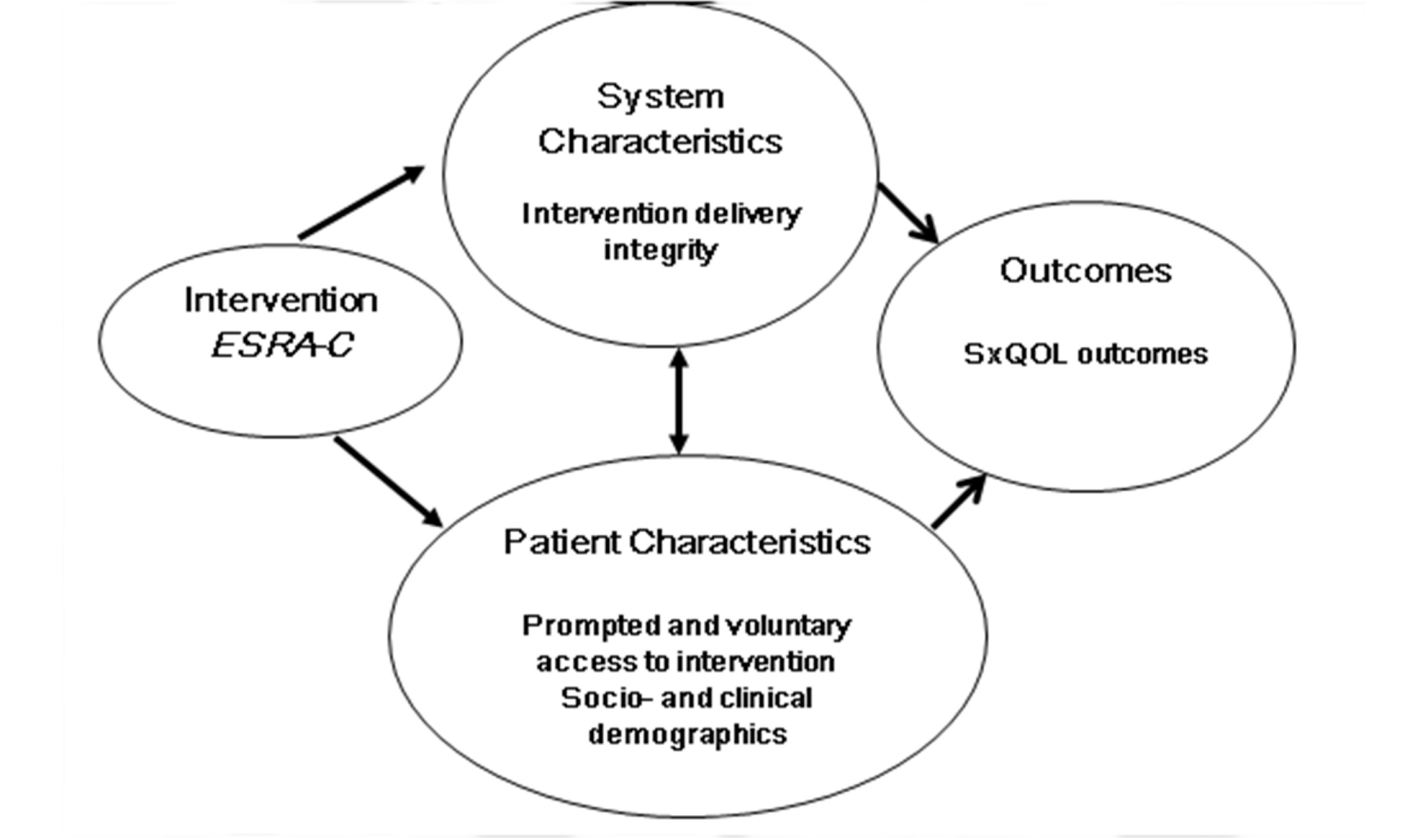
Adapted Health Outcomes Model [10].

This analysis addresses one component of our program of research founded on the Quality Health Outcomes Model, a framework proposed by Mitchell and colleagues [10] to illustrate that patient outcomes are rarely explained only by specific interventions but also by health care system/provider factors and patient-specific factors. The extent of patients’ use of the intervention can be placed in the model (Figure 1) as a patient-specific factor that may influence the impact of the ESRA-C intervention on symptom distress.

### Design, Sample, Intervention

The ESRA-C II was a randomized trial conducted at two comprehensive cancer centers. Full details of the trial [5] and system development were reported elsewhere [11]. In brief, adult participants with any type or stage of cancer, and about to start a new anti-cancer therapy, used ESRA-C to self-report baseline SxQOL and then were randomized to receive usual education about SxQOL topics or usual education plus the opportunity to self-monitor when not in the clinic, tailored self-care instruction for SxQOL issues, and communication coaching on how to report each SxQOL to clinicians. While in the clinic, the intervention group participants were shown an overview of the ESRA-C intervention and voluntary remote use was encouraged. Participants in the intervention group could access the ESRA-C program from home or in clinic on a touch-screen computer at any time throughout the trial to electronically track SxQOL and view the intervention. Those intervention group participants without Internet access were encouraged to meet the research coordinator during any subsequent visit to the clinic and use ESRA-C on a study tablet. Participants in both groups were asked to report SxQOL using the ESRA-C system from home or clinic at three study time points (T2-T4) throughout the course of therapy, coinciding with clinic visits at which clinicians would receive a printed summary of the patient report for participants in both groups. Home user participants in both groups were prompted by email, 24 hours prior to a scheduled clinic visit, to use the SxQOL report feature of ESRA-C. Clinic users were notified to arrive about 45 minutes prior to scheduled clinic visits corresponding to each study time point in order to use the reporting feature and, if in the intervention group, components. Intervention group participants had access to the ESRA-C intervention *Teaching Tips* and *View My Reports* components.

**Figure 2:**
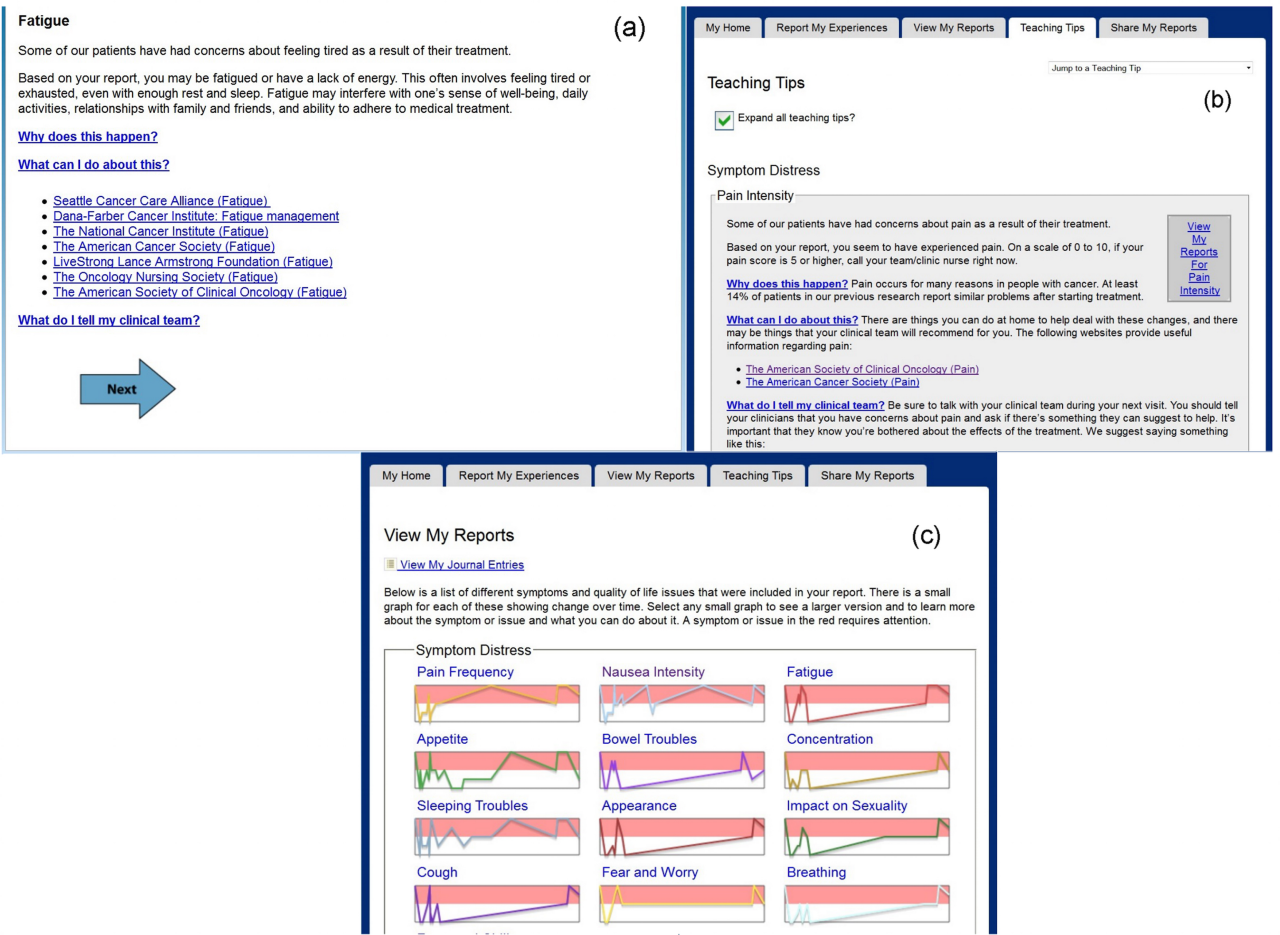
Exemplar screen shots from the ESRA-C intervention: a) pushed Teaching Tip, b) Teaching Tips tab, c) View My Reports tab.

Following the SxQOL report in prompted T2-T4 sessions, the intervention group participants received pushed teaching tips for those SxQOL issues reported as moderate-to-severe. Within each pushed teaching tip was the option to expand linked text addressing (1) “Why does this happen?”, (2) “What can I do about this?”, and (3) “What do I tell my clinical team?” (Figure 2a). After the SxQOL report and pushed teaching tips, the participant could navigate to the *Teaching Tips* tab or the *View My Reports* tab within the intervention home page (Figure 2b-c) by clicking on the designated tab. A click on the (non-pushed) *Teaching Tips* tab displayed a dropdown list of all 26 SxQOL issues and the option to select and expand any issues. A click on the *View My Reports* tab displayed thumbnail line graphs tracking SxQOL reports over time.

Intervention group participants were invited to access ESRA-C at any time between prompted sessions and clinic visits. These sessions were defined as any intervention use that was not prompted. During voluntary, non-prompted sessions, the participant did not receive pushed *Teaching Tips*, but did have the option to report SxQOL, and click the (non-pushed) *Teaching Tips* tab and the *View My Reports* tab.

The ESRA-C intervention was considered delivered if the participant accessed the *Teaching Tips* and/or *View My Reports*. As a conservative measure of exposure to *Teaching Tips*, if at least one pushed tip was delivered during a prompted session, this was considered comparable to a single click on the non-pushed *Teaching Tips.* For example, a participant with at least one pushed teaching tip at each of three prompted sessions would have a total of three pushed teaching tips. Total exposure to the intervention consisted of three components: (1) the number of pushed teaching tips during prompted sessions, (2) the number of clicks on the non-pushed Teaching Tips tab during prompted and non-prompted sessions, and (3) the number of clicks on the *View My Reports* tab during prompted and non-prompted sessions. Voluntary exposure only occurred during non-prompted sessions and consisted of (1) the number of clicks on the (non-pushed) *Teaching Tips* tab, and (2) the number of clicks on the *View My Reports* tab.

At prompted study time points, all participants were presented a set of SxQOL self-report assessments that included the Symptom Distress Scale-15 (SDS-15) [5], cancer quality of life questionnaires EORTC QLQ-C30 [12] and EORTC-CPIN20 [13], the Patient Health Questionnaire (PHQ-9) depression scale [14], a 0-10 pain intensity numerical scale, and a skin problems questionnaire [15]. At unprompted sessions, intervention group participants could choose to access any or all of the questionnaires. These procedures were fully described previously [5].

A total of 752 participants were randomized in the parent trial: 374 intervention and 378 control. Of those, 523 (262 intervention, 261 control) had complete SDS-15 baseline and end-of-study scores. In the primary analysis of covariance (ANCOVA), the average SDS-15 score was reduced by an estimated 1.21 points (95% CI 0.23-2.20; *P*=.02) in the intervention group compared to the control group [5].

### Analytic Methods

**Figure 3:**
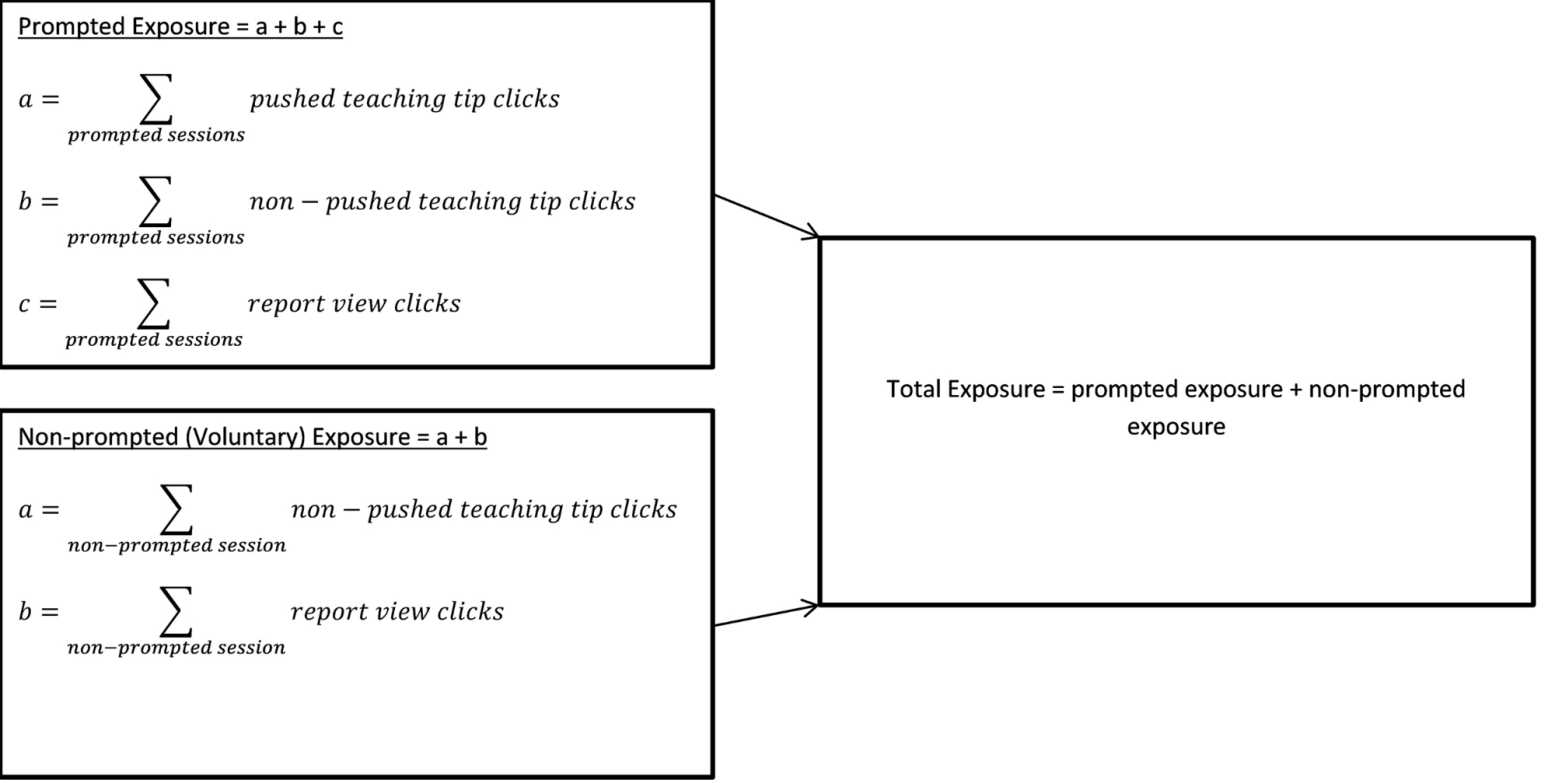
Components of the ESRA-C intervention with calculation of total exposure.

Total exposure was calculated as the sum of the number of clicks on pushed teaching tips, clicks on the non-pushed *Teaching Tips* tab, and the number of clicks on the *View My Reports* tab. Similarly, voluntary exposure was calculated as the sum of the number of clicks on the *Teaching Tips* tab and the number of clicks on the *View My Reports* tab during non-prompted sessions (Figure 3). Descriptive statistics were used to summarize the total and voluntary intervention exposures. The median of total exposure was calculated and used to indicate sufficient exposure to the intervention and defines intervention use.

Factors associated with using the intervention, both overall and voluntarily, were explored with Fisher’s exact test. Factors of interest included: age (≥50 years, <50 years), work status, frequent computer user, gender, married/partnered, education (>high school, ≤high school), and service (medical oncology, radiation oncology, and stem cell transplant). Stage of disease was not considered for two reasons: (1) the highly associated relationship of stage and working status, a phenomenon documented in our prior work [16], and (2) the study sample contained participants with hematologic cancers in which the solid tumor staging system was inappropriate. The propensity score [17-19] was used to match a subset of the control group to the exposed intervention group and was defined as the probability of using the intervention given baseline participant characteristics. The following factors, shown in prior work [5] to contribute to outcomes, were used to compute the propensity score: baseline SDS-15 score categories (15-19, 20-23, 24-28, >28), service, gender, frequent computer use, married/coupled, education, minority status, age category, and working status. The outcome, change in continuous SDS-15 scores from pre-treatment to end of study, was compared by intervention use and selected by propensity score matching using an ANCOVA approach.

Propensity score matching was performed with the complete data method in which no missing information could exist in the covariates used to compute the propensity score. A sensitivity analysis was conducted with the goal to balance the missingness within the two most common missing factors, work status and minority status. The propensity score matched sample was obtained using the R package “MatchIt” [20] and nearest neighbor matching. All analyses were performed in SAS version 9.3 and R version 2.15.2. All tests were two-sided and considered significant at the .05 level.

## Results

### Intervention Group Analysis

Total and voluntary exposure to the intervention components can only be calculated within the intervention group. The median total exposure was 2 (range 0-29). Intervention use was defined as at least two exposures. Of the 374 intervention participants, 233 (62.3%) used the intervention. A software error precluded exposure to pushed teaching tips for any intervention participants with an appointment date from June 30, 2010 to May 12, 2011. Of the 141 not receiving pushed teaching tips in the intervention, this error precluded three possible exposures in 48 (34.0%), two exposures in 21 (14.9%), and 1 exposure in 15 (10.6%) intervention group participants. There were 55 participants on the intervention arm that required a clinic/assisted point of access; their median total exposure was 2 (range 0-13) and 16 (29%) viewed the intervention voluntarily in the clinic. There were 319 participants on the intervention arm that indicated home/independent access; the median total exposure for these remote users was 3 (range 0-29) and 111 (34.8%) viewed the intervention voluntarily.

There were no statistically significant differences in the proportion of participants using versus not using the intervention based on characteristics, with the exception of clinical service (*P*=.02, Table 1). A total of 70.4% (88/125) of radiation oncology participants, followed by 60.2% (127/211) of medical oncology participants, used the intervention, whereas only 47% (18/38) of transplant participants used the intervention. The median voluntary exposure to the intervention was 0 (range 0-16). Voluntary use was defined as at least one voluntary exposure. Of the 374 participants randomized to the intervention group, 127 (34.0%) voluntarily used the intervention. There were marginally significant differences in the proportion of participants voluntarily using the intervention by work status (*P*=.06) and education (*P*=.05). Participants that used the intervention were working and had more than a high school education. Additionally, there was a significant difference in the proportion of participants voluntarily using the intervention by service (*P*=.001). More radiation oncology participants 58/125 (46.4%) voluntarily used the intervention compared to medical oncology 61/211 (28.9%) and transplant 8/38 (21%) participants (Table 1).

**Table 1:** Number and frequency of total exposures and voluntary exposures by selected participant characteristic.

Characteristic		Overall n	Total exposure (at least 2)		Voluntary exposure (at least 1)	
			n (%)	*P* value	n (%)	*P* value
Total N		374	233 (62.3)	*-*	127 (34.0)	*-*
**Age**				1.00		.82
	≥50 years	248	154 (62.1)		83 (33.5)	
	<50 years	126	79 (62.7)		44 (34.9)	
**Work status**				.64		.06
	Working	222	141 (63.5)		84 (37.8)	
	Not working	123	75 (61.0)		34 (27.6)	
**Frequent computer use**				.24		.07
	No	59	33 (55.9)		14 (23.7)	
	Yes	304	195 (64.1)		111 (36.5)	
**Gender**				.13		.33
	Male	185	108 (58.4)		58 (31.3)	
	Female	189	125 (66.1)		69 (36.5)	
**Minority**				.86		1.00
	No	304	190 (62.5)		104 (34.2)	
	Yes	36	22 (61.1)		12 (33.3)	
**Married/Partnered**				.79		.14
	No	78	48 (61.5)		21 (26.9)	
	Yes	293	185 (63.1)		106 (36.2)	
**Education**				.10		.05
	≤High school	71	38 (53.5)		17 (23.9)	
	>High school	301	195 (64.8)		110 (36.5)	
**Service**				.02		.001
	Medical oncology	211	127 (60.1)		61 (28.9)	
	Radiation oncology	125	88 (70.4)		58 (46.4)	
	Hematopoietic stem cell transplant	38	18 (47.4)		8 (21.0)	

### Propensity Score Analysis

**Figure 4:**
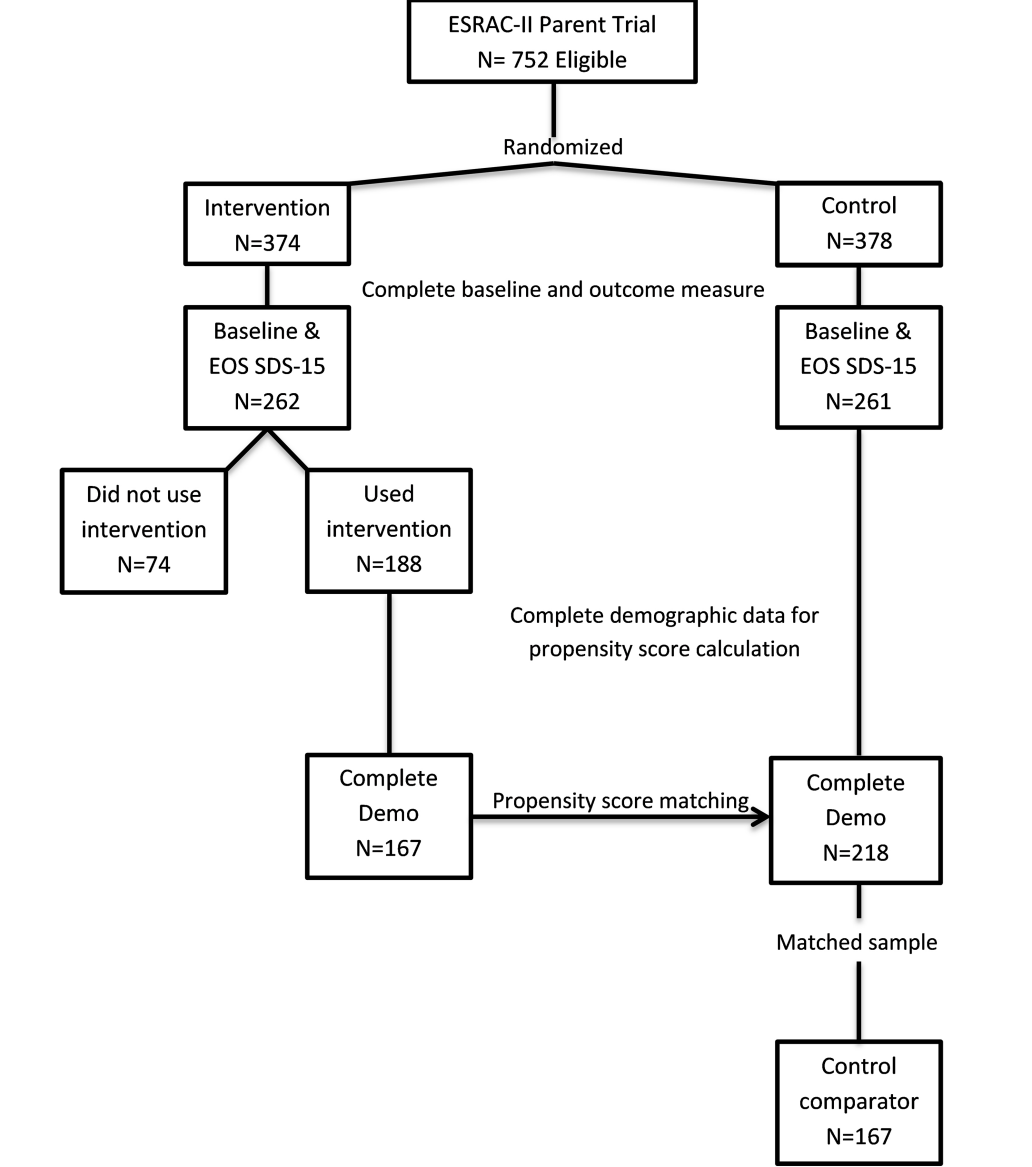
Sample selection for the propensity score analysis. Note: EOS=end of study; SDS=Symptom Distress Scale; Demo=demographics.

Figure 4 outlines the sample selection from the parent trial for the propensity score analysis. Of the 262 participants randomized to the intervention group with a baseline and end-of-study SDS-15 score, 188 (71.8%) used the intervention. Complete demographic data were available for 167 (88.8%) of the 188 who used the intervention and 218 (83.5%) of the 261 control participants with baseline and end-of-study SDS-15 score. Using the propensity score and nearest neighbor matching, 167 control participants were selected from the possible 218 as the matched control group. Covariates were confirmed to be balanced (data not shown). Participants who used the intervention had lower symptom distress; mean change in the SDS-15 score was 1.07 (SD 6.55) in the matched control group (higher distress) and −0.57 (SD 5.68) in the intervention group (lower distress). In the ANCOVA analysis, SDS-15 score was reduced by an estimated 1.53 points (95% CI 0.32-2.75; *P*=.01) in the intervention group compared to the matched control group. The sensitivity analysis that balanced the missingness within the work status and minority status factors produced similar results as the complete data analysis (data not shown).

## Discussion

### Principal Findings

The results of this propensity analysis suggested that reduction of cancer symptom distress, the primary outcome of our randomized trial, was associated with use of the ESRA-C intervention components. More than half of participants in the intervention group were exposed to pushed *Teaching Tips*, accessed non-pushed tips, and viewed reports of SxQOL outcomes graphed over time. About a third voluntarily accessed the intervention in between clinic visits. We discovered that use of the intervention significantly reduced the estimated symptom distress score when compared to participants who did not use the intervention. The magnitude of the estimate (1.53) was larger than in the primary outcome analysis when we compared study groups (1.21) [5], indicating that actual use promotes the impact of the intervention. While this may seem intuitive, actual use of psycho-educational or self-administered interventions is not always known to investigators without objective monitoring capability. Our findings are consistent with the Quality Health Outcomes Model [10], illustrating the influence of patients’ characteristics.

### Comparisons With Prior Work

Use of, and adherence to, Web-based health care interventions have primarily been evaluated in health promotion and chronic disease self-management settings [21]. Few studies have associated changes in physical and/or psychosocial symptom distress with use of an eHealth intervention by patients with cancer and none for patients undergoing active cancer therapies or for those in the United States. Borosund et al [22] analyzed usage of a Web-based, symptom distress self-management system by prostate and breast cancer survivors in Norway over one year post-enrollment. Similar to our analysis, the Norwegian group defined “use” as at least two intervention sessions, but did not analyze symptom outcomes based on use. Van der Berg and colleagues [7] analyzed usage statistics of a Web-based self-management intervention for breast cancer survivors in the Netherlands. Participants were prompted by email to access the intervention. The survivors did not monitor or report symptoms, but were encouraged to read and/or view new educational material provided every 4 weeks over 16 weeks. Active usage was defined by Ven den Berg's group as a log-in to each of the four modules, and was observed in 44% of the 70 women in the trial. Our unprompted voluntary use percentage was lower at 34% of 374 intervention group participants. The explanation for a lower voluntary use percentage may be related to no systematic prompting for voluntary use or the fact that, unlike the group of Dutch survivors, our participants already were receiving the intervention at three time points prior to clinic visits throughout active cancer therapy.

Not surprisingly, ESRA-C was accessed remotely and voluntarily more frequently by those with higher education, who may have been likely to use personal computers or tablets on a regular basis. This is consistent with the finding, while of borderline statistical significance, that working individuals also used ESRA-C more often in between clinic visits. Working when beginning cancer therapy has been shown as a significant variable in two of our earlier analyses, predicting a lower rate of emergency department and unplanned admissions [23], yet predicting depression in participants receiving stem cell transplant [24]. How the fact that a patient is working full- or part-time influences outcomes is not well understood. In this case however, participants who were working when about to start cancer therapy may have had the type of job that enabled easy access to the Internet.

Participants who enrolled in the trial as they were about to undergo radiation treatments also accessed ESRA-C voluntarily significantly more often than those enrolled when beginning medical cancer therapies or stem cell transplant. We are not aware of differences in usual care symptom support between modality services at the cancer centers; yet, if differences existed, patients may have turned to ESRA-C more often in radiation. Alternatively, these participants were reminded of ESRA-C almost every day of the week as they entered the radiation setting where each had consented to the trial.

### Limitations

Our findings are limited by the fact that about a third of the intervention group participants never received pushed teaching tips in the assessments prior to on-study clinic visits. Thus, the effect of the intervention may have been different if all had the opportunity to see the tips. Our participant sample was less diverse with regard to race and ethnicity than the rates of cancer diagnoses in minority groups in the United States [25] and all were patients at comprehensive cancer centers, precluding generalization of our findings beyond these parameters.

### Implications for Future Research

First, propensity analyses could be replicated in other eHealth trials as a method to investigate the relationship of usage to health outcomes. Although our participants’ raw exposure to the intervention was not high in an absolute sense, we were able to study the association of symptom distress with usage rather than report raw usage. Second, investigators could evaluate whether usage was related to various characteristics, and whether they are characteristics of the intervention, of the user, or of the condition addressed by the intervention. We provided some rationale for the mechanism that triggered usage by certain demographic groups, but this could have been a combination of aspects of the intervention itself in addition to participant demographics. Although some investigators found that educated, older, employed women were the most active users of Web-based, chronic disease [26] and health promotion [27] interventions, other studies have revealed conflicting results with regard to demographic variables [28-29].

### Implications for Clinical Practice

Our findings have implications for the many patients treated at institutions that have deployed a patient portal as a component of an electronic medical record system. There may be patients at risk for failed symptom and distress screening and/or failed symptom support delivery if such systems are available only to those Web-savvy, educated patients who regularly use email.

Finally, implications for both future research and practice using patient-centered, Web-based technologies include improved communication of study design and workflow between the design and technical implementation teams and more rigorous quality checks on intervention integrity. Communication of research goals may be facilitated via improved use of shared artifacts such as models of study data and workflows [30]. Software unit-testing and continuous integration goals should be oriented toward detailed research data deliverables [31,32]. Methods to improve quality checks include improved training of software quality assurance staff and making descriptive study data available to the investigators early in the data collection period for interim review.

### Conclusions

The intended effects of a Web-based, self-care education, monitoring, and communication coaching intervention on cancer symptom distress were modified by intervention use frequency. The voluntary, remote use of ESRA-C was most frequent in working participants with higher education levels and those receiving radiation therapy.

## Abbreviations

ANCOVA: analysis of covariance
ESRA-C: Electronic Self Report Assessment for Cancer
SDS-15: Symptom Distress Scale
SxQOL: symptoms and quality of life
